# New records and modelling the impacts of climate change on the black-tailed marmosets

**DOI:** 10.1371/journal.pone.0256270

**Published:** 2021-09-07

**Authors:** Almério Câmara Gusmão, Jôine Cariele Evangelista-Vale, João Carlos Pires-Oliveira, Adrian A. Barnett, Odair Diogo da Silva

**Affiliations:** 1 Programa de Pós-Graduação em Biotecnologia e Biodiversidade, Rede Bionorte, Universidade do Estado de Mato Grosso, Cáceres, Mato Grosso, Brazil; 2 Programa de Pós-Graduação em Desenvolvimento Sustentável, Centro de Desenvolvimento Sustentável, Universidade de Brasília—UnB, Brasília, DF, Brazil; 3 Programa de Pós-Graduação em Ecologia, Universidade do Estado de Mato Grosso, Nova Xavantina, Mato Grosso, Brazil; 4 Department of Zoology, Federal University of Pernambuco, Recife, Pernambuco, Brazil; 5 Amazon Mammals Research Group, National Institute for Amazonian Research, Instituto Nacional de Pesquisas da Amazônia, Manaus, AM, Brazil; 6 Department of Life Sciences, Roehampton University, London, United Kingdom; 7 Programa de Pós-Graduação em Ciências Ambientais, Centro de Pesquisa de Limnologia, Biodiversidade, Etnobiologia do Pantanal, Universidade do Estado de Mato Grosso, Cáceres, Mato Grosso, Brazil; Universidade Federal de Mato Grosso do Sul, BRAZIL

## Abstract

Climate change represents an unprecedented threat to global biodiversity and, for many species, gaps in our knowledge of their biology remain acute. Gaps in baseline knowledge, such as confirmed identifications (Linnean shortfalls) and adequate collections (Wallacean shortfalls), need to be minimized with new studies, since this is often critical for effective conservation. Despite the increase in scientific research on primates in the southwest of the Brazilian Amazon, little is known about the species *Mico nigriceps* (Ferrari & Lopes, 1992) Primates, Platirryni. In the current study, we sought to reduce the extent of the Wallacean shortfall for *M*. *nigriceps*, understand whether climate change represents a threat to the distribution of the species, and identify priority areas for its conservation. Accordingly, we provide 121 new records in 14 locations, obtained directly from the field, and five from the literature. Using this, we carried out ecological niche modeling, to better understand how environmental suitability might limit the area occupied by the species. We then projected a distribution for 2070 with the SSP2-4.5 (more optimistic) and SSP5-8.5 (more pessimistic) scenarios. Our data confirmed the geographic distribution of the species as being restricted to headwaters of the Ji-Paraná/Machado river, but with a 400 km extension to the south. Under the modeled climate change scenarios, the area suitable for the species declines by 21% under the most optimistic, and by 27% in the pessimistic, scenario across the projected 50-year period. Although we have expanded the area of known occurrence for this species, we point out that climate change threatens the stability of this newly-discovered population strongly, and that this danger is intensified by deforestation, fire and hunting. We recommend that further studies be carried out to confirm the presence of the species in adjacent areas, those indicated by generated models as being potential environmentally suitable. In addition, we recommend intensifying forest restoration in currently pastured areas, and protection of the areas forming the current and future habitat of this species through such measures as protected area creation.

## Introduction

Threats to global biodiversity have reached unparalleled levels, with the majority caused directly or indirectly by human activities [1, 2]. Since they threaten the survival of taxonomic groups in diverse ecosystems, such occurrences represent an unprecedented challenge for conservationists planet-wide [3–7]. Prominent among these is climate change, which is being accelerated by industrial activities and changes in land use [3].

While the impacts of climate change on scientifically-described species are alarming, their potential impact on species for which knowledge is still limited is also of great concern. Such losses are incalculable due to the many knowledge gaps in terrestrial biodiversity. This includes gaps in such basic information as the abundance (Prestonian shortfalls), evolutionary patterns (Darwinian shortfalls), limits of species traits (Raunkieran shortfalls), form of biotic interaction (Eltonian shortfalls), abiotic tolerances of species (Hutchinsonian shortfalls),species distribution patterns (Wallacean shortfalls), and the taxonomic delimitation of species (Linnean shortfalls) [8, 9]. Such gaps present a particular challenge for those who wish to understand species distribution patterns so as to develop effective conservation strategies [10]. Such issues are aggravated in megadiverse regions, such as the tropics, where species richness per unit area is greatest, but overall knowledge is poor [3].

Basic questions of taxonomy and distribution exist even for high-profile groups such as primates, especially for smaller-bodied species. For Neotropical primates, for example, despite the increase in the general number of studies, the species *Mico nigriceps* [11] (Primates: Callitrichidae) remains one of the least-known primates, with a lack of even basic information, such as geographic distribution and general ecology [11]. The original description of *M*. *nigriceps* [11] indicated the geographic distribution was unknown in its entirety, with only two locations being confirmed in the Madeira-Marmelos interfluvium, on the mid-section of the Madeira river, in the Brazilian states of Amazonas and Rondônia. Prior to this, *Mico* populations from the head of the Ji-Paraná/Machado river had been allocated to *M*. *emiliae* [12], based on studies of specimens collected during studies by the Integrated Development Program of Northwest Brazil (POLONOROESTE). These were collected in the district of Nova Brasília (today the municipality of Ministro Andreazza, Rondonia State), and their taxonomic allocation was followed by subsequent studies [11, 13–15]. *Mico nigriceps* has the general color pattern characteristic of the *argentatus* group [11]: the body is silvery-gray, with the coat darkening on the posterior and ventral parts of the body; the dark pigmentation of the face and ears is a diagnostic characteristic of the species; the general pattern is for black extremities; the arm, mantle and belly have light brown and orange tones, the back is brown, and the hind limbs brownish-orange; the tail is completely black [11].

Nearly three decades after its scientific description, *M*. *nigriceps* is still little-known scientifically. Current knowledge of the species is limited to that given by Ferrari and Lopes [11], plus subsequent additional data-points [16]. Accordingly, an expansion of knowledge of the species, its ecology and true distribution, thus minimizing the effects of Wallacean and Linnean shortfalls, is highly desirable, especially given that the species appears on the Brazilian List of Endangered Species as Data Deficient (DD) [17]. In addition, the extent of habitat destruction in the region where the species occurs has been severe, especially in recent years, as policies of the incumbent Brazilian government have resulted in a loosening of environmental law enforcement, and enhanced natural habitat degradation [18].

In this context, Ecological Niche Modeling (ENM) can be used to guide sampling efforts. The ENM produces layers of range of species distribution, using the environmental variables of locations with known occurrence records, that is, allows determination to a species her environmental suitability area (ESA). This can, by extrapolation, be used to identify new sites where occurrence is likely, thus helping to minimize the effects of any Wallacean Shortfall [19]. In addition, ENM makes it possible to project into the future the distribution of species suitability under different climate change scenarios. This allows assessment of the extent and nature of the effects of climate change on the distribution of the target species, making it highly relevant for guiding biodiversity conservation policies, such as measures aimed at creating and maintaining conservation units [20, 21]. As a result, ENM is a useful scientific tool for improving knowledge of little-known species, such as *M*. *nigriceps*, as well as enabling quantified estimates of the degree of threat that such species will face under various climate change regimes.

In the current study we have used a new set of field-derived records and ENM to achieve the following objectives: 1) expand the known distribution area of the species *Mico nigriceps*; 2) Identify locations with highest potential environmental suitability for this species; and 3) to understand whether climate change will affect the extent and distribution of areas environmentally suitable for this species, considering the predictions for the year 2070.

## Materials and methods

### Study area

**Fig 1:**
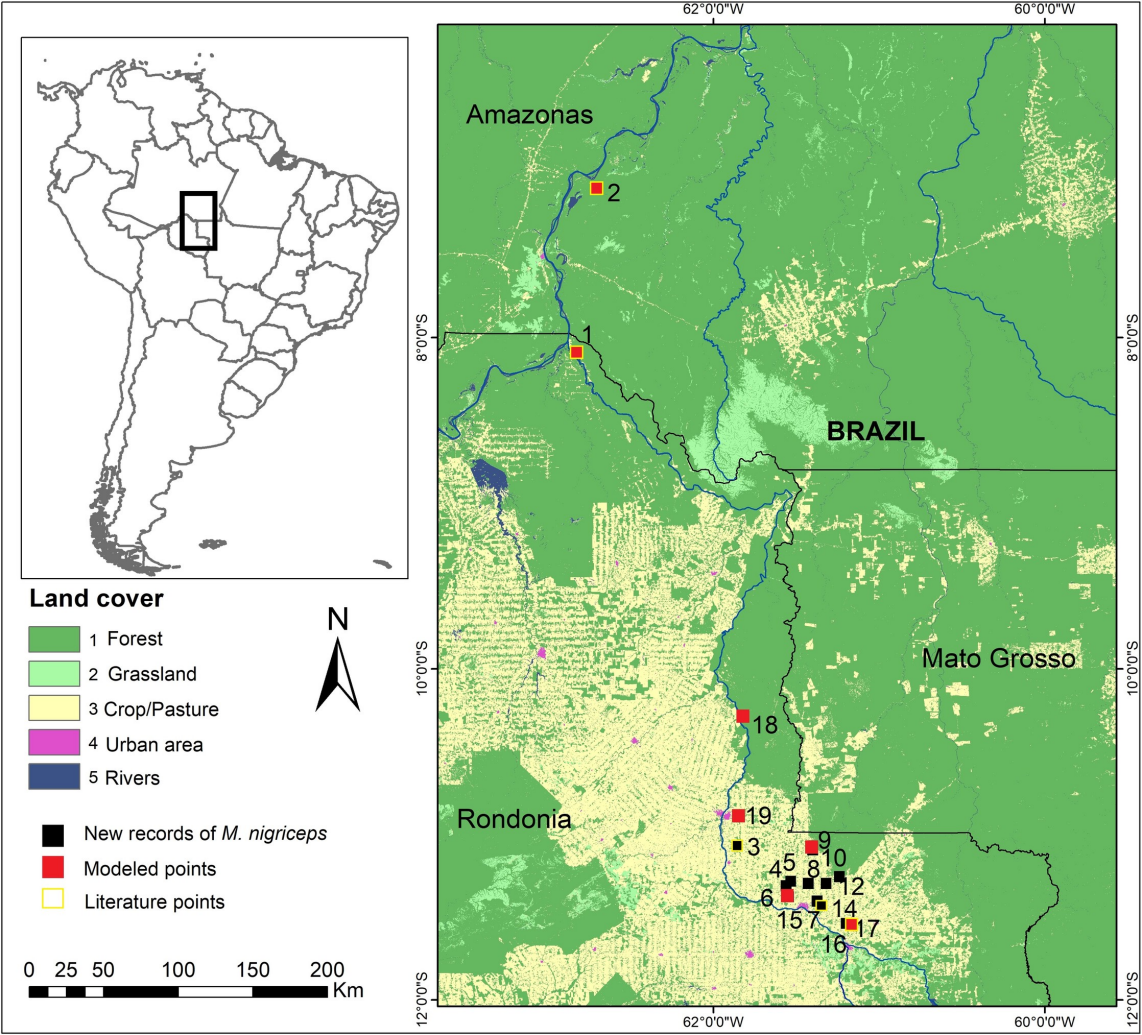
Study area map. Map of southwestern Brazilian Amazon showing new records of *M*. *nigriceps*, and the other known records of species of the genus *Mico*. The records for *M*. *rondoni* were extracted from [24, 25]; *M*. *melanurus* records are from [26, 27]; *M*. *intermedius* and *M*. *marcai* records were extracted from [28]. Reprinted from Souza et al. (10.3390/rs12172735) under a CC BY license, with permission from MapBiomas project, original copyright 2019. MapBiomas Project—Collection 5 of the Annual Series of Coverage and Land Use Maps in Brazil, accessed on 04/14/2021 through the link: https://mapbiomas.org/colecoes-mapbiomas-1?cama_set_language=pt-BR. Shapefiles by: Ministério de Meio Ambiente.

We collected *M*. *nigriceps* location records via direct field sampling efforts in an area located between the middle Ji-Paraná/Machado and Comemoração rivers (Fig 1). Sampling occurred between June 2008 and January 2020. The regional phytophysiognomy is of Open Rainforest [22], containing terra firme (never-flooded) and floodplain (igapó) forest forms throughout. The climate is of the tropical AW type (warm and humid), with a dry season during the coldest months (June to September), and a rainy season during the warmer months (December to March), with an average temperature of 26°C [23].

### Occurrence records

The new *M*. *nigriceps* records were obtained during five expeditions: a rapid inventory of primates at Fazenda Cajazeiras, carried out between June 20, 2008 to June 29, 2008, “Line 2”, Cacoal, Rondônia, Brazil, totaling 72 hours of sampling effort; rapid mastofaunal inventory of Sítio Laranjeiras, carried out on February 12 and 13, 2009, “Line 10”, Cacoal, Rondônia, totaling 16 hours of sampling effort; a survey in the Jaru Biological Reserve, from October 28 to 30, 2016, in the city of Ji-Paraná, Rondônia, totaling 26 h of sampling effort. Other records occurred opportunistically during travel within the region.

To identify the species, we photographed the animals encountered during field studies, and collected four specimens found as road-kill. These specimens were taxidermized and deposited in the Mastozoologica Collection of the Limnology, Biodiversity and Ethnobiology Research Center of the Pantanal, Mato Grosso State University, Cáceres, Mato Grosso, Brazil, (CELBE-M-ODS-002; CELBE-M-ODS -003; CELBE-M-ODS-010 and CELBE-M-ACG-1399). They were used for comparison with the illustrations and diagnosis of *M*. *nigriceps* described in Ferrari & Lopes (1992) [11], and images of skins available in Garbino (2014) [28]. We also compared them with skins of the species deposited at the Museu Paraense Emílio Goeldi (MPEG 22960; MPEG 22962). Comparison of museum collection specimens, and the photographs of the encountered animals, confirmed the species identification. We used the occurrence records available in the literature, and the new records obtained through this study to generate ecological niche models.

Brazilian law requires that the collection of dead animals, such as those collected in this study, require prior authorization from the responsible environmental agency (Normative Instruction 03/2014, Article 25, paragraph 3). According to this legislation, we obtained a permanent license for the collection of zoological material (Number: 6825–1) from the Chico Mendes Institute for Biodiversity Conservation—ICMBio, on behalf of the Museu Paraense Emilio Goeldi, through the Biodiversity Authorization and Information System—SISBIO. Thus, for the execution of this research and the eventual disclosure of data, we are supported ethically and legally.

As specimens studied were road-kill victims, ethics committee permission to sacrifice the animals was not required since, according to Brazilian legislation, this kind of collection does not require other legal documents, just the possession of a generalized license for the collection of zoological material.

### Ecological Niche Modeling (ENM)

#### Environmental variables

The literature recommends a cautious approach to choosing the size of the area to be analysed, as this can greatly affect the performance of the models [29, 30]. Size of chosen area must also be commensurate with study aims: accordingly, our models were generated for the entire neotropical region [31], since we aimed to identify all new suitable sites with for the target species, so using a more circumscribed area could limit the identification of such areas.

To produce the ENM, we obtained 19 bioclimatic variables from the *WolrdClim 2*.*0* [32]database. We also used ten edaphic and topographic variables, that is, six variables with soil physical properties (depth to bedrock, bulk density, clay, coarse, sand, and silt) from SoilGrids database (https://soilgrids.org/) and three topographic variable: elevation, obtained from the EarthEnv database (http://www.earthenv.org/); and aspect and slope, obtained using the *terrain* function of the raster package from R [33, 34] (see S1 Table in S1 File). The variables were clipped for the neotropical region using a shapefile mask (available at http://ecoregions2017.appspot.com/), and then were resampled at a 5-minute arc-sine resolution (~ 10k).

To test for collinearity between the selected variables, we performed two Principal Component Analysis (PCA). PCA 1 was generated for the bioclimatic variables, from which we selected the first six axes as model predictors, since these explained ~ 95% of data set variation over the neotropical region. We performed a second PCA (PCA 2) for the edaphic and topographic variables, selecting the first two axes, which represented 66% of the variation within the same region. We attributed less weight to the variables in this second PCA because bioclimatic variables form the central focus of the current study.

We generated projections for the bioclimatic variables, to 2070, under two different greenhouse gas emission scenarios generated by the Intergovernmental Panel on Climate Change (IPCC): a more optimistic scenario (SSP2-4.5), and the more pessimistic (SSP5-8.5). We chose 2070 as the period in which to understand the effects of climate change on the distribution of this species to provide an extensive, but still tangible, period of time, and to reduce the effects of uncertainties arising from climate projections beyond 2090 [4].

These simulated variables were produced based on the projections of the Coupled Model Intercomparison Project Phase 6 (CMIP6) [33]. The CMIP6 provides different scenarios based on the socio-economic pathway chosen (SSP) [34]. We did not run a new PCA on the variables of the future, as this would cause a loss of correspondence between the PCA axes produced for the current and the future variables. Using the *PCA Projection* function of the R package *ENMGadgets* [5], we produced a PCA using current variables, and designed the linear coefficients of the current PCA using the future variables. This controlled for the percentage of explanatory power of each variable for each PCA axis in both the present and the future without losing correspondence between the PCAs [35].

In a similar manner, the ten edaphic and topographic variables were summarized on PCA axes, using a Broken-Stick model to select the number of axes to be included in each model. According to the premises of Broken-Stick modeling, the PCA axes selected, were the two that explained more of the variation than the null model produced by the analysis. Since there are no temporal projections of edaphic and topographic variables, the two more informative components selected by the broken-stick criterion were used in all climate scenarios, since we do not expect geological processes to act in such a short time. On the other hand, such variables are important in modeling [36], and we chose to include them, but used the same edaphic and topographic PCAs axes created with the present-day variables to build the projections of environmental suitability for all present and future changes scenarios used in this study [37].

#### Modeling

For modeling the following algorithms were used using the R biomod2 package, following [38]. We used nine algorithms: Generalized Boosted Models (GBM), Classificatory Tree Analysis (CTA), *Random Forest* (RF), Generalized Linear Model (GLM), Generalized Additive Model (GAM), Artificial Neural Networks (ANN), Flexible Discriminant Analysis (FDA), Multiple Regression Adapted for *Splines* (MARS) and *Maximum Entropy* (MAXENT).

During modeling, *M*. *nigriceps* occurrence records were tallied using a gradient system based on cells 10km X 10km per side. We did not obtain a minimum of 25 spatially unique occurrence records, that is, a single record in a cell of 100km², which would add at least 25 cells to the grid with an occurrence record in each. Due to the small amount of data available, we used the Jackknife approach for training and evaluating model performances [39, 40]. In this approach, an occurrence record is removed from the modeling and the capacity of the model to predict the existence of the removed locality is tested. We repeated the procedure of removing an occurrence record until all points had been excluded from the modeling at least once, and then evaluated performance with a binomial test, which showed whether our model was better than one produced at random [40].

Most modeling algorithms require data with presence and absence records for the modeled species. However, confirmed absence data is rare for most species, and in these cases we used pseudo-absences (PAs). PAs are random points selected within the study area background that serve to represent absences when calibrating and evaluating models. We calibrated each algorithm using the *biomod2* package default configuration, as this modifies only pseudo-absence numbers (PAs). In such cases, the PAs in GBM, RF and CTA were ranked based on the number of occurrences, as recommended by [41]. For GLM, GAM, ANN, FDA, MARS and MAXENT models, 10000 PAs points were randomly selected, following [42]. In addition, PAs were generated using the two sets of algorithms via a *disk* arrangement, which defined the minimum and maximum distances at all points of occurrence available for the species, with PAs sampled within these minimum and maximum limits, that is, inside the “disk” [38]. A set of 10 PAs was run in 10 modeling sequences for each set of PAs, equivalent to 100 models for each algorithm [39].

After removing an occurrence record, producing the models and obtaining the maps with the continuous projections of each algorithm, we joined the projections of all algorithms in a single continuous averaged ensemble map. We performed this procedure every time we removed an occurrence record. After obtaining the continuous modeling maps, we built binary representation maps, where 0 indicates absence and 1 presence of the species. To produce such binary maps, we used the threshold that maximizes the sum of sensitivity and specificity, commonly called MaxSpecSens or ROC Threshold [42], so that all cells in the study area that had suitability values above the threshold were considered as presence (value 1), while all study area values with suitability less than the threshold were marked as species absent (value 0).

Finally, we combined all the binary maps that matched the points left out of the modeling into a final consensus map that represented the most suitable conditions for *M*. *nigriceps*. We did this for the present and for the two future climate change scenarios. All maps were edited with ArcGIS 10.5 software.

## Results

**Fig 2:**
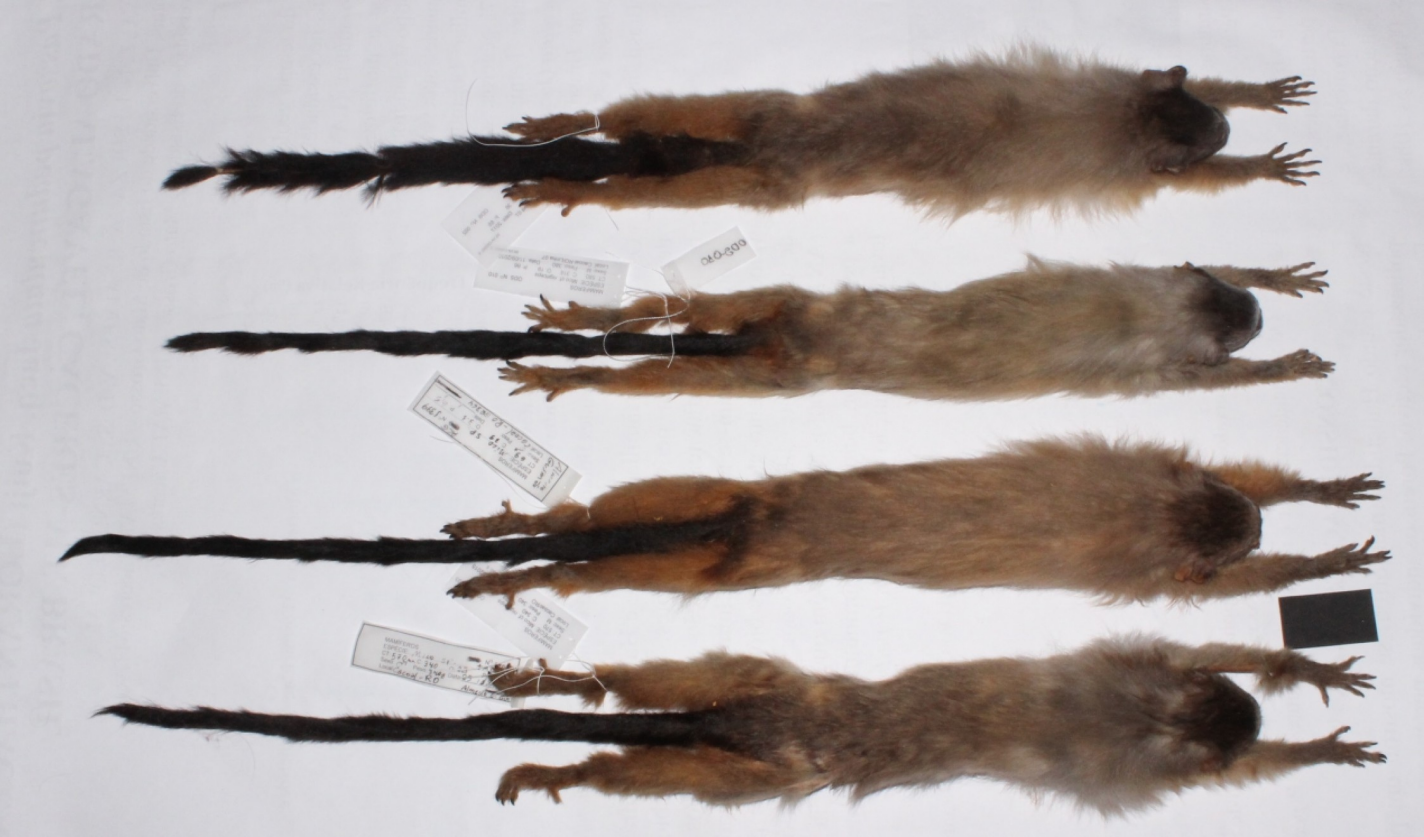
Analyzed skins. Road-kill derived specimens deposited in the Mastozoologia collection, CELBE Pantanal, and analysed for the current study (Photo: O. Silva-Diogo).

In total, 121 individuals of *M*. *nigriceps* were recorded across the 14 field locations studied (Fig 2 and S2 Table in S1 File). To this were added five records available from the literature. However, due to the close proximity of the locations from which the occurrence records were obtained, we had only seven spatially unique occurrence points, that is, distributed individually in 10kmx10km each side cells to generate the models.

**Fig 3:**
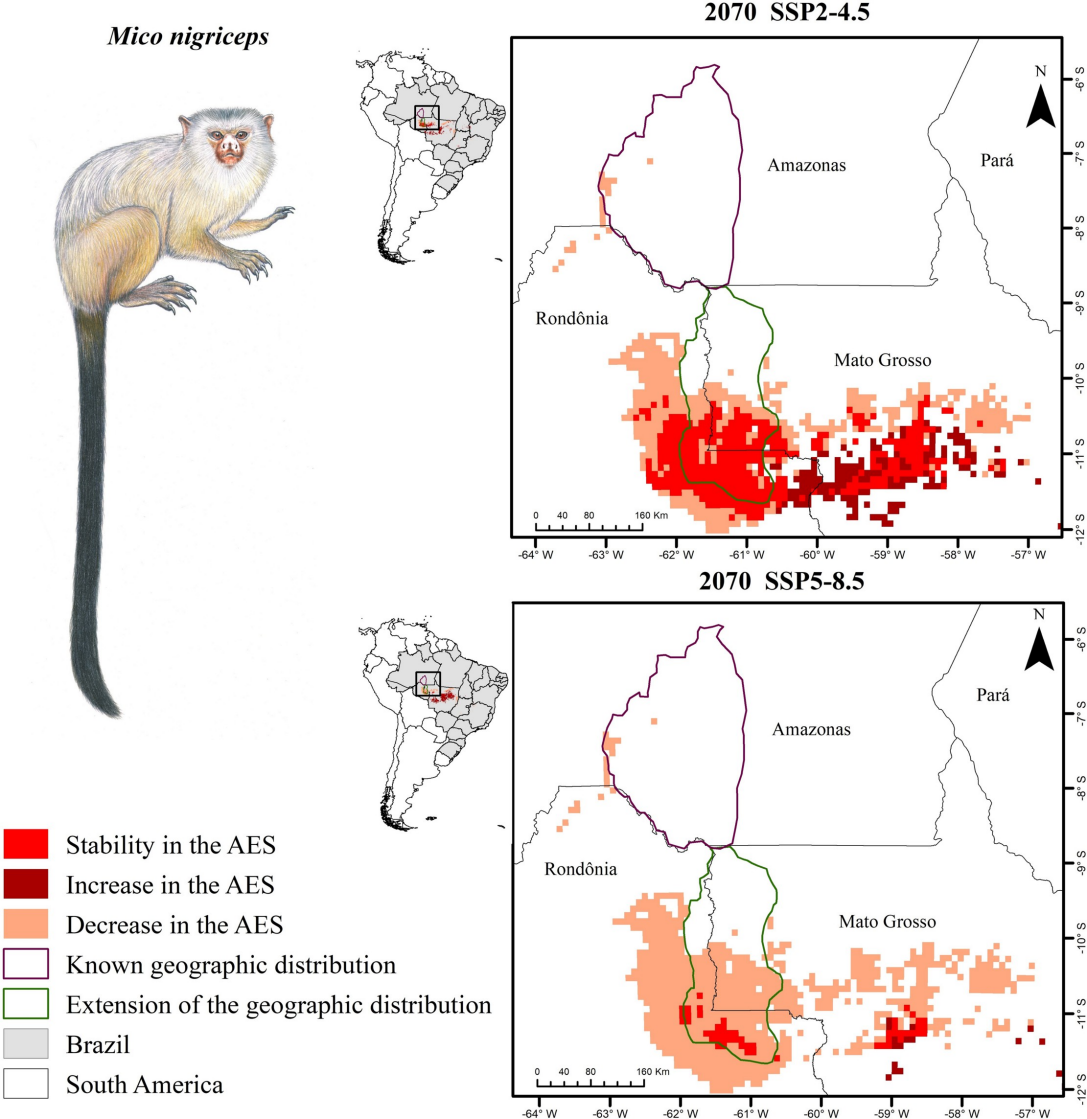
Modeling-generated map. Modeling-generated map of current and future distributions for *M*. *nigriceps*. *Mico nigriceps* illustration reprinted from Ferrari and Lopes (1992) under a CC BY license, with permission from Stephen D. Nash, original copyright 2021. Shapefiles by: Ministério de Meio Ambiente. AES Area.

The new set of records, obtained for the species in the current study, reveal a range that is far more extensive than known previously, occupying an area from the mouth of the Ji-Paraná/Machado river to its headwaters and west of the upper Roosevelt river (S2 Table in S1 File). This, limited to the south by a non-forested grassland-covered plateau. The conducted ecological niche modeling analysis performed better than random (p> 0.0001). Overall, ENM indicates that, under the current climate scenario, the area of environmental suitability for the species occupies a large swathe of the neotropics (Fig 3), with models indicating that the AES for the species occurs both in the original area of occurrence, and in the region covered by the new records presented in the current study (Fig 3). Based on projections for 2070 using currently predicted climate change scenarios, the species will lose 100% of AES in its historically-known distribution under all run scenarios. Under SSP2-4.5, the most optimistic scenario, the species AES will decline 30.4%; while in the most pessimistic scenario (SSP5-8.5), the AES loss was modeled as 85.11% of the current AES for the species.

The results also indicate the presence of *M*. *nigriceps* in protected areas (PAs) and indigenous land (IL) (S2 Table in S1 File) under the current climate regimen, both for the geographic range as originally described (282.83 km²), as well as for the newly-expanded distributional area reported by the current study (252,978.69 km²). In total, under the current climatic regime 7.25% of the expected area of ​occurrence of the species overlaps with protected areas. However, most of the distribution of the species area (~82.8%) overlaps with areas under pasture and on private properties. In the future, this scenario worsens, as AES for *M*. *nigriceps* in PAs and ILs is predicted to occur only in the area of occurrence added by the current study, declining to 4,552.96 km² in the SSP2-4.5 scenario and 32.39 km² in the SSP5-8.5 scenario. Both climate change scenarios indicate that there will be AES for this species only in IL Sete de Setembro (Table 1).

**Table 1:** Protected areas (PAs) and indigenous lands (ILs) with AES for *M*. *nigriceps* in current climate scenario, and under the scenarios SSP2-4.5 and SSP5-8.5 (expressed as km²).

Histórical occurence area			
	Current climate	SSP-4.5	SSP-8.5
PA Nova Aurora	18.52	-	-
PA Gibeão	31.29	-	-
PA Irmão Satelis	41.09	-	-
PA Jaru	125.76	-	-
IL Roosevelt	141.65	141.65	-
IL Igarapé Lourdes	1,816.87	605.88	-
IL Zoró	2,656.59	1,575.40	-
IL Sete de Setembro	248,146.92	2,230.04	32.39
**Total**	**152,978.69**	**4,552.96**	**32.39**

## Discussion

The results obtained during this study considerably expanded the number of known *M*. *nigriceps* records, which enabled to know better the distribution of the species and to model its AES. When the geographic distribution limits of *M*. *nigriceps* were defined by [11], it included the area east of the Ji-Paraná/Machado and Madeira rivers, and west of the Marmelos river. The southern limit was described as a plateau of non-forested land that separates the head of the Marmelos river from the right bank of the Ji-Paraná river. Consequently, the new occurrence records have provided evidence of a range extension of the distribution of more than 400 km south. This more than doubles the extent of the scientifically-established distribution of the species, and can be considered as a good approximation of the area of *M*. *nigriceps*´s distribution. The confirmation of the presence of species to the south of its formerly known range is of great importance, precisely because it is an area where a large area of native forest (and hence suitable habitat) still remains.

The models shows that the known area of distribution of the species [11] occupies only a small part of the potential AES indicated by the ENM [11]. The ENM indicated adjacent areas with AES potential for the species, which highlights the need for studies in these new areas to confirm these predictions [9]. Results also suggest that the type locality may occupy a peripheral portion of the actual geographic range of the species. This reinforces the important contribution that ENM can make to rare species conservation studies, since the models generated under the present climate scenario indicate an area of environmental suitability for the species in the areas where our field surveys confirmed actual presence. Thus, this tool can be useful for guiding future studies on other species whose true distribution is little known.

Our results also indicate that climate change will have a negative impact on the area of occurrence of this species, both that historically-known and the area newly-added by the current study, especially in the most pessimistic scenario, SSP5-8.5. Under both optimistic and pessimistic climate change scenarios, AES losses in the previously-known area of occurrence [11] will be 100%. Additionally, it will also be very severe in both the new area of distribution described in this study, and in the areas adjacent areas indicated by ENM as likely to harbor the species. Even under the most optimistic scenario, losses to the *M*. *nigriceps* AES will be significant. This reinforces the opinion that biodiversity in the southern Amazon region will be strongly impacted by changes in climate [43], with the current study acting as a bellwether for this.

Modeling points to other work that the climate change in the neotropical region has been affecting the distribution of low mobility mammal species since the last glacial maximum [44]. In the future, this trend could to aggravate associated with deforestation that can take approximately 25% of mammalian species to lose> 40% of their current habitat [45]. This is especially worrying for neotropical primates that lives in trees. Thus, due the changes in environmental conditions, enhanced by the intensification of highly-impactful human activities [46] the stability of primates populations is also at risk, as already discussed for *Ateles chamek*, *Lagothrix cana* and *Leontopithecus chrysopygys* [47–50], mainly in the Amazon / Cerrado Ecotone Region. The probability of a decrease in the occurrence of these species is intensified under future climate change scenarios, since the habitat fragmentation associated with changes in climate conditions strongly threatens population stability, as well as with interaction networks with other populations [46].

Therefore, climate change threatens the existence of species like *M*. *nigriceps*, whose historical shortfalls in identification and sampling have been remedied only recently. The results of the current study highlight the need to intensify field studies so that conservation actions can be prepared for other species in similar situations. For these, ENM can assist in identifying both study areas, and priority sites for the effective conservation of such species [50]. For *M*. *nigriceps*, the loss of an AES within its current range represents a threat to the existence of the local population, which can be a prelude to the extinction of the species as a whole [51].

As a member of an iconic taxon, the primates, measures to conserve *M*. *nicriceps* species would act as an umbrella for the protection of many others in the region. Accordingly, we suggest efforts are concentrated in areas with the greatest suitability for such species, especially those which contain native vegetation, but which do not yet have legal status as protected area. Private areas with native forests are also important for species conservation. For this, investing in land use planning must be a priority, since they can establish such areas as Legal Reserve and Environmental Preservation Areas, as governed by Brazilian legislation. This should be both studied and have protection extended to them. We emphasize the necessity of intensifying conservation efforts linked to this species, since the area that modeling indicates as environmentally appropriate for it is located in the Brazilian Amazon “arc of deforestation”.

The biodiversity of this region has been severely impacted by human actions resulting from the expansion of export-related agricultural activities, in addition to deforestation, which has increased exponentially in recent years [52] and recent fires, mainly in the years 2019 and 2020 [52]. These actions have been intensified by the weakening of environmental protection policies in Brazil, especially in the Amazon [53]. This increases the need to carry out reforestation activities in these areas, as well as in areas used as pastures. While this should occur in priority areas, we would also suggest this action be implemented in adjacent areas, with lower possibility of predicted future occurrence. We suggest this since it both provides protection to the priority areas, via buffering, and strengthens the connectivity matrix between the forest fragments, enabling long-term population viability.

Although climate change may seem to be a distant threat, the pressure from human activities may lead to the end of the occurrence of *M*. *nigriceps*, and many associated species, in the region in a very short period of time, and in a few decades they will be able to overcome the impacts of deforestation [54]. Climate change is an additional impact on this primate, as well as other species, in a region already under severe anthropic pressure. Furthermore, despite the conservation potential the protected areas represent [55], our projections for the two future climate change scenarios considered indicates that the environmental suitability for the species in both these areas will decrease. This may be related to the intensification of the effects of climate change in the southern part of the Amazon [34]. This demonstrates that not even the presence of protected areas will be sufficient to effectively conserve this species, since human actions increasingly threaten the integrity of the biodiversity they currently protect.

We suggest that *M*. *nigriceps* be a co-beneficiary in the case of conservation of carbon stocks, since the stimulus will occur in the conservation of Protected Areas and priority areas on private lands. This will be most pronounced in the case of forest restoration in regions with potential distribution of the species in the south of the species distribution, where the greatest loss of habitat is concentrated. However, as has been shown analytically [56] prioritizing the carbon stock alone does not meet the conservation goals of a species. This suggests that, to achieve these goals, investments must simultaneously follow the objectives of conservation of the species and conservation of carbon stocks.

## Conclusion

Our work demonstrates the need for a broad sampling of species occurrence sites in order to better understand species distribution, as this allows for more accurate model calibration, which is useful to better plan species conservation strategies. In addition, we highlight the risks inherent in the deficient knowledge of the range of distribution of species, which can lead to imprecise conservation strategies. Finally, we warn of the risks arising from climate change on *M*. *nigriceps*, because, if the change scenarios are confirmed, the range of suitability for the species studied here, which is currently ~ 82,027km², may suffer losses of 30.4% - 85.11%. Of the total predicted, only 7.25% in fact are protected in protected areas, which makes urgent actions essential to minimize the effects of climate change, as well as the creation of management and recovery strategies for areas that have been converted into pastures so that we can preserve both *M*. *nigriceps* and other species in the community that co-occur with it.

## Supporting information

S1 File (DOC)
