# Supplementary material for: New records and modelling the impacts of climate change on the black-tailed marmosets
Source: PLoS One. 2021 Sep 7;16(9):e0256270. doi: 10.1371/journal.pone.0256270 (PMC8423304; doi:10.1371/journal.pone.0256270)
Supplement: S1 File — (DOC) [file pone.0256270.s001.doc]

**S1 Table.** Environmental variables used in the modeling of *M. nigriceps.*

| **Environmental Variables** | **Sources** |
| --- | --- |
| Annual Mean Temperature | WORLDCLIM 2 [1] |
| Mean Diurnal Range | WORLDCLIM 2 [1] |
| Isothermality | WORLDCLIM 2 [1] |
| Temperature Seasonality | WORLDCLIM 2 [1] |
| Max Temperature of Warmest Month | WORLDCLIM 2 [1] |
| Min Temperature of Coldest Month | WORLDCLIM 2 [1] |
| Temperature Annual Range | WORLDCLIM 2 [1] |
| Mean Temperature of Wettest Quarter | WORLDCLIM 2 [1] |
| Mean Temperature of Driest Quarter | WORLDCLIM 2 [1] |
| Mean Temperature of Warmest Quarter | WORLDCLIM 2 [1] |
| Mean Temperature of Coldest Quarter | WORLDCLIM 2 [1] |
| Annual Precipitation | WORLDCLIM 2 [1] |
| Precipitation of Wettest Month | WORLDCLIM 2 [1] |
| Precipitation of Driest Month | WORLDCLIM 2 [1] |
| Precipitation Seasonality | WORLDCLIM 2 [1] |
| Precipitation of Wettest Quarter | WORLDCLIM 2 [1] |
| Precipitation of Driest Quarter | WORLDCLIM 2 [1] |
| Precipitation of Warmest Quarter | WORLDCLIM 2 [1] |
| Precipitation of Coldest Quarter | WORLDCLIM 2 [1] |
| Elevation | EARTHENV [2] |
| Slope | *terrain* function from *raster* package [3] |
| Aspect | *terrain* function from *raster* package [3] |
| Bulk density (mean 0.0 m - 0.30 m) | SOIL GRIDS [4] |
| Clay content (mean 0.0 m - 0.30 m) | SOIL GRIDS [4] |
| BDRICM-M: depth to bedrock (r horizon) up to 200 cm | SOIL GRIDS [4] |
| Sand content (mean 0.0 m - 0.30 m) | SOIL GRIDS [4] |
| Coarse fragments % | SOIL GRIDS [4] |
| Silt content (mean 0.0 m - 0.30 m) | SOIL GRIDS [4] |

**S2 Table.** Localities with records of *M. nigriceps* in south-west Amazonian Brazil.

| Site | Locality | | Geographical coordinates | N° ind. obs. | Groups/10km | Reference |
| --- | --- | --- | --- | --- | --- | --- |
| 1 | | Calama (Porto Velho, RO) | 7°31’00”S 62°52’00”W | - | - | [5] |
| 2 | | Lago dos Reis (Humaitá, AM) | 08°03’00”S 62°53’00”W | - | - | [5] |
| 3 | | Nova Brasília | 11°09’00”S 61°34’00”W | - | - | [6] |
| 4 | | Fazenda Cajazeiras | 11°18’33”S 61°38’41”W | 23 | - | New record |
| 5 | | Sitio São Jorge | 11º15’09”S 61º35’40”W | - | - | [7] |
| 6 | | Urban area (Cacoal,RO) | 11°25’06”S 61°28’05”W | 14 | - | New record |
| 7 | | Urban area (Cacoal,RO) | 11°26’28”S 61°26’18”W | 7 | - | New record |
| 8 | | Sitio Odair | 11°18’53”S 61°26’30”W | 21 | - | New record |
| 9 | | TI Sete de Setembro | 11°12’24”S 61°11’02”W | 3 | - | New record |
| 10 | | Linha 8 (Cacoal, RO) | 11°23’13”S 61°24’16”W | 15 | - | New record |
| 11 | | Linha 9 (Cacoal, RO) | 11°29’47”S 61°22’22”W | 6 | - | New record |
| 12 | | Linha 10 (Cacoal, RO) | 11°17’14”S 61°19’44”W | 7 | - | New record |
| 13 | | BR 364 (Cacoal, RO) | 11°27’28”S 61°25’17”W | 1 | - | New record |
| 14 | | APP rio Pirarara (Cacoal, RO) | 11°25’43”S 61°26’37”O | 12 | - | New record |
| 15 | | APP rio Tamarupá (Cacoal, RO) | 11°25’40”S 61°28’01”O | 16 | - | New record |
| 16 | | BR 364 (Pimenta Bueno) | 11°35’37”S 61°14’44”O | 1 | - | New record |
| 17 | | Sitio Lagoa Azul (Pimenta Bueno, RO) | 11°36’25”S 61°09’54”O | - | 2.1 | [8] |
| 18 | | Linha 86, km 27, Nova Colina, RO | 10°49’38”S 61°32’18”O | 5 | - | New record |
| 19 | | Reserva Biológica do Jaru (Jí-Paraná, RO) | 10°00’18”S 61°58’25”O | 5 | - | New record |

**References**

1. Amatulli G, Domisch S, Parmentier B,Ranipeta A, Malczyk J, Jetz W. A suite of global, cross-scale topographic variables for environmental and biodiversity modeling. **Scientific Data**, 2018. 5, 1, 180040.
2. Fick SE, Hijmans RJ, WorldClim 2: new 1-km spatial resolution climate surfaces for global land areas. International Journal of Climatology, . 2017. 37, 12, p. 4302–4315, 1.
3. Hijmans RJ. raster: Geographic Data Analysis and Modeling, 2018. Disponível em: <https://cran.r-project.org/package=raster>
4. Hengl T, Jesus JM, MacMilian RA, Batijes NH, Heuvelink GBM, Ribeiro E, Samuel-Rosa A, Kempen B, Leenaars JGB, Walsh MG, Gonzales MR. SoilGrids1km – Global soil information based on Automated mapping. PlosOne 2014. <https://doi.org/10.1371/journal.pone.0105992>
5. Ferrari SF, Lopes MA. New data on the distribution of primates in the region of the Jiparana and Madeira River in Amazonas and Rondônia, Brazil. Goeldiana Zool. 1992; 11:2-12.
6. Vivo M. On some monkeys from Rondônia, Brazil (Primates: Callitrichidae, Cebidae). Papéis Avulsos de Zoologia, Museu de Zoologia da Universidade de São Paulo. 1985; 36:103-110.
7. Oliveira LS. Dieta de sagui da Amazônia (*Mico* ssp.) em um fragment de floresta, no município de Ministro Andreazza, Rondônia – Brasil. Trabalho de Conclusão de Curso. Faculdade de Ciências Biomédicas de Cacoal. 2009.
8. Monção GR. Estimativa populacional e abundância de primatas em um fragmento de florestal no município de Pimenta Bueno, Rondônia – Brasil. (Trabalho de conclusão de curso) Faculdade de Ciências Biomédicas de Cacoal. Facimed. 2008.
